# Postoperative recurrence of diffuse sclerosing thyroid cancer in an adolescent patient: A case report

**DOI:** 10.1097/MD.0000000000037246

**Published:** 2024-02-16

**Authors:** Ningning Ren, Chong Geng, Kailin Liu, Jintao Ren, Xuan Zhang, Xingsong Tian

**Affiliations:** a Shandong University, Cheeloo College of Medicine, Jinan, Shandong, China; b Department of Breast and Thyroid Surgery, Shandong Provincial Hospital, Jinan, Shandong, China; c Department of Breast and Thyroid Surgery, Shandong Provincial Hospital Affiliated to Shandong First Medical University, Jinan, Shandong, China; d Minquan County People’s Hospital, Shangqiu, Henan, China; e The Second Hospital of Shandong University, Jinan, Shandong, China.

**Keywords:** case report, diffuse sclerosing variant, papillary thyroid carcinoma, recurrence and metastasis

## Abstract

**Background:**

Papillary thyroid cancer is an inert malignant tumor with a good response to surgical treatment, low recurrence and metastasis rate, and good prognosis. Diffuse sclerosing thyroid cancer is an invasive subtype that is more common in young people, with a higher rate of lymph node metastasis and recurrence, and a relatively poor prognosis.

**Patient concerns:**

A 13-year-old girl underwent radical surgery for diffuse sclerosing thyroid cancer. Eight years later, due to a large number of lymph node metastases, she underwent another radical surgery on her neck lymph nodes.

**Methods:**

The patient thyroid ultrasound and neck enhanced CT indicated that the patient had multiple enlarged lymph nodes in the neck with irregular morphology and structure, and the possibility of metastatic lymph nodes was high. Subsequently, the patient underwent thyroid fine-needle aspiration and the results showed that cancer cells were detected in both cervical lymph nodes.

**Diagnosis:**

The patient was diagnosed with bilateral cervical lymph node metastases after thyroid surgery.

**Results:**

After the second surgery, the patient recovered well, and no residual or focal iodine uptake tissue was found on the enhanced CT examination.

**Conclusion:**

As diffuse sclerosing thyroid cancer is prone to lymph node and recurrent metastases, once it is diagnosed, radical treatment should be actively performed. Postoperative adjuvant radiation therapy should be administered according to the patient condition and regular follow-ups should be conducted to monitor neck lymph node metastasis.

## 1. Introduction

Thyroid cancer is a common endocrine malignancy with papillary thyroid carcinoma (PTC) being the most common type. Overall, the main characteristics of PTC are low recurrence and metastasis rates, and good prognosis. Diffuse sclerosing variant of PTC (DSV-PTC) is a special subtype of PTC. The incidence rate thereof is low, but malignancy is relatively high, recurrence and metastasis rates are high, and prognosis is relatively poor.^[1]^ We report the case of a young patient with DSV-PTC who experienced postoperative recurrence. We followed this patient for 8 years to provide assistance and reference for the clinical treatment of DSV-PTC.

## 2. Case presentation

### 2.1. Patient information

**Figure 1.**
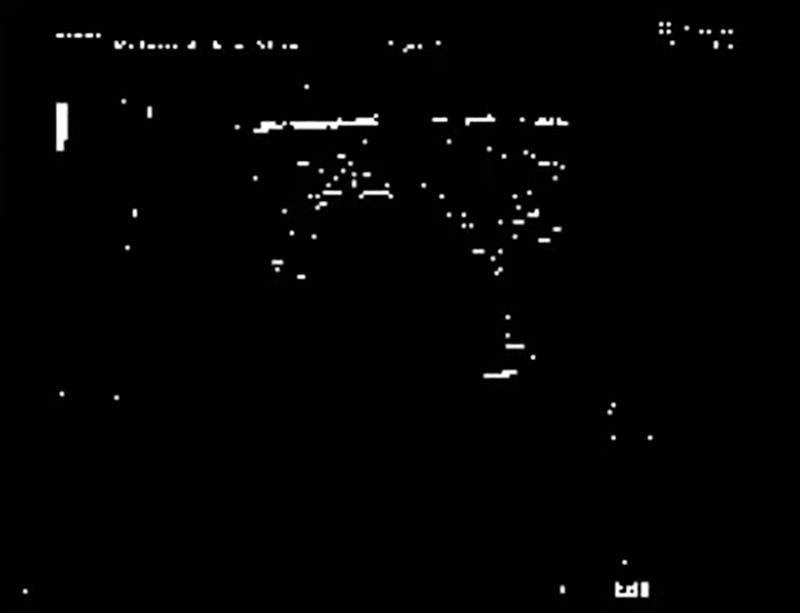
Diffuse distribution of punctate strong echoes in the thyroid gland involving the entire lobe of the gland (due to age, the image quality may be poor).

**Figure 2.**
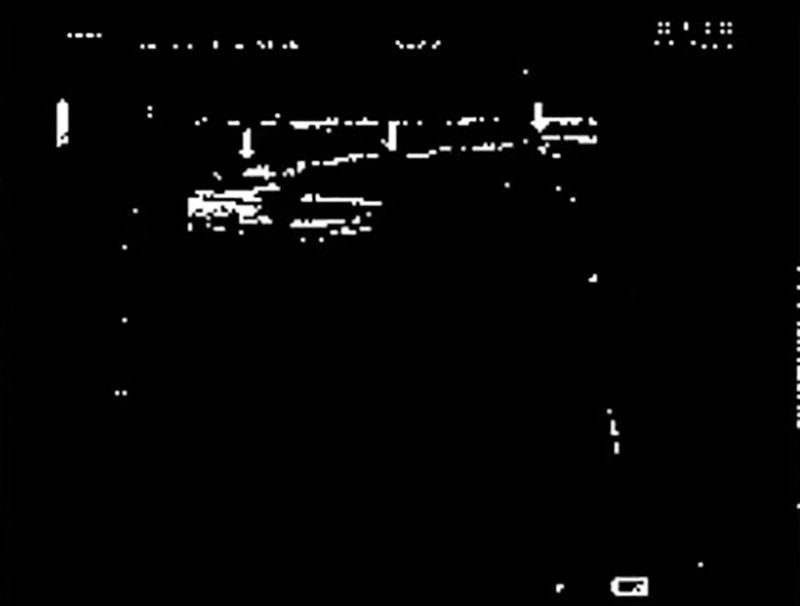
Multiple lymph nodes can be seen in the neck with unclear structures (due to age, image quality may be poor).

A 13-year-old female patient was admitted to the hospital in 2015 for a physical examination of a thyroid enlargement she had been experiencing for more than half a month. She had no symptoms, such as hoarseness, coughing due to drinking water, or difficulty in breathing or swallowing. She was previously healthy without any other accompanying diseases. There is no related family history either. Since the onset of the disease, the patient had been mentally and physically stable, eating and sleeping well, urinating normally, and there was no significant change in weight. Physical examination and palpation revealed a second-degree enlargement of the thyroid gland, with a hard texture and poor mobility. The patient free triiodothyronine, free thyroid hormone, and anti-thyroid peroxidase antibody levels were normal. Thyroid-stimulating hormone levels were slightly elevated (4.85 μIU/mL); and anti-thyroglobulin antibody levels were > 500 IU/mL, which is significantly higher than the normal value, indicating the presence of Hashimoto thyroiditis (HT). Preoperative ultrasound findings of the patient are shown in Figures 1 and 2. Diffuse distribution of punctate strong echoes were observed in the thyroid gland, involving the entire lobe of the gland, with a denser left lobe and isthmus. In addition, multiple lymph node echoes were detected around the trachea, with the largest being approximately 0.8 × 0.5 cm, with a plump shape and unclear structure, with some visible solid echoes and strong echogenic light spots. Multiple lymph nodes were detected in the II to IV area of the bilateral neck, with the larger one on the right located in the IV area, with a size of approximately 2.1 × 0.8 cm, and the larger one on the left located in Zone IV, with a size of approximately 1.9 × 0.6 cm, with unclear structure and visible patchy high echoes. Preoperative laryngoscopy revealed no obvious abnormalities, indicating that the tumor had not invaded the trachea. Chest radiography revealed no obvious abnormalities, indicating that the tumor had not invaded the lungs.

The patient subsequently underwent surgical treatment, removing all thyroid glands and clearing the bilateral neck lymph nodes in areas II, III, IV, V, and VI. Intraoperative frozen pathology suggested a poorly differentiated thyroid adenocarcinoma with diffuse infiltrative growth and local papillary carcinoma. Postoperative paraffin pathology revealed DC-PTC (in the left lobe, isthmus, and right lobe), with cancer cells found in 6/16 cervical lymph nodes. Immunohistochemical results were as follows: CK19 + (strong), Galectin-3+, CK8/18+, and CA125 + (focal). The patient was discharged on the 6th day after surgery. At discharge, the patient was generally in good condition. The patient was prescribed 100 μg oral levothyroxine sodium tablets (to be taken on an empty stomach) every day. A follow-up of thyroid function was performed 1 month after surgery and medication dosage was adjusted based on thyroid function results. Two months after surgery, the patient received I131 treatment at a dose of 110 mCi. Subsequently regular thyroid ultrasound examinations showed no obvious abnormalities.

### 2.2. History of current disease

**Figure 3.**
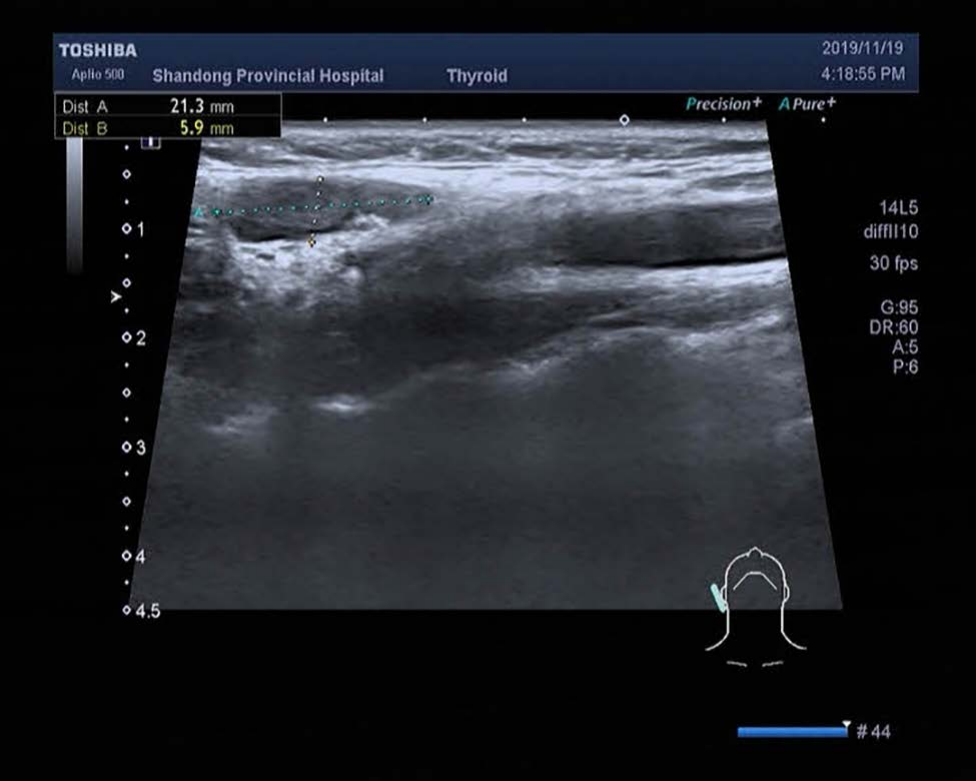
Right region II lymph node, approximately 2.1 × 0.6 cm in size, multiple dotted strong echoes can be seen.

**Figure 4.**
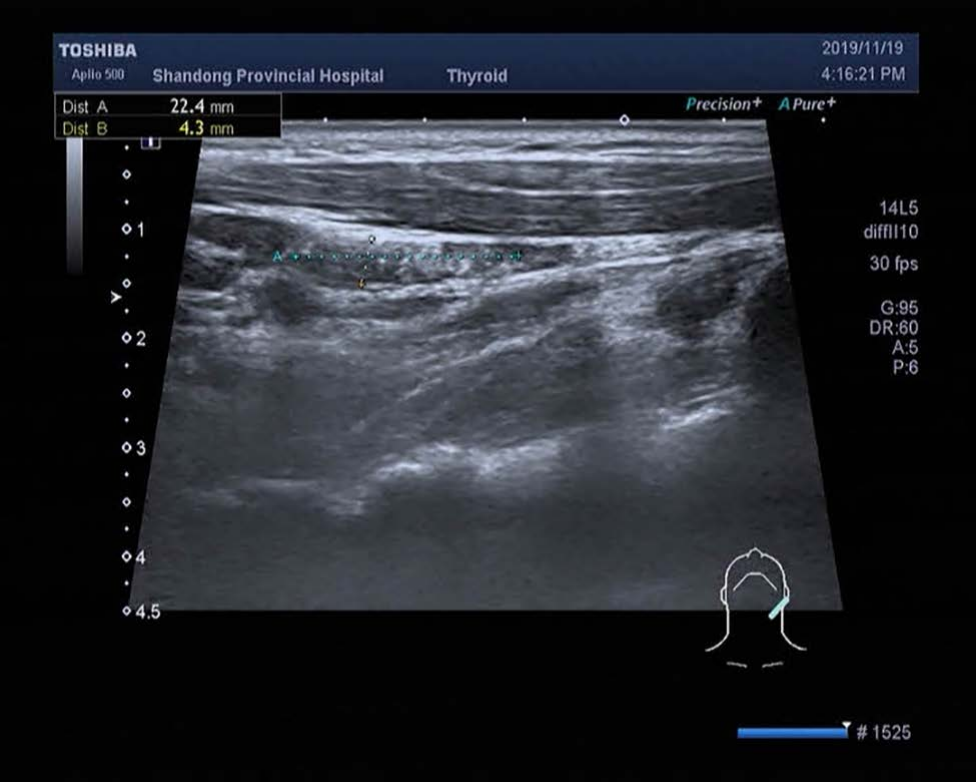
Right region III lymph node, approximately 2.2 × 0.4 cm in size, multiple punctate strong echoes can be seen.

In 2019, 4 years after the surgery, thyroid ultrasound showed several lymph node echoes were detected around the trachea, with the largest being approximately 1.1 × 0.7 cm in size, with an unclear corticomedullary boundary and multiple punctate strong echoes visible in the cortex (Figs. 3 and 4). Several lymph nodes were detected in both regions II and III of the neck on both sides, with the larger one on the right located in region II, with a size of approximately 2.1 × 0.6 cm, and the larger one on the left located in Zone III, with a size of approximately 2.2 × 0.4 cm; multiple punctate strong echoes could be seen. Considering the high possibility of recurrence in these lymph nodes and the patient unwillingness to undergo further surgical treatment, we advised the patient to undergo iodine 131 treatment again with regular follow-up, with the understanding that if any changes occurred, prompt medical attention should be sought.

**Figure 5.**
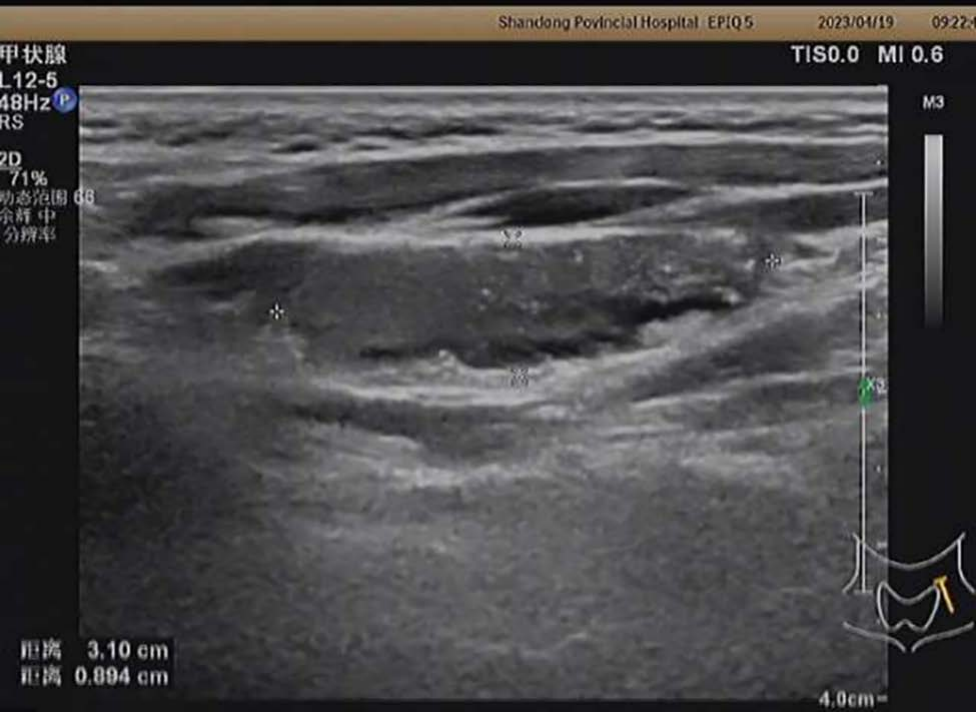
Left region III lymph nodes have uneven echoes and speckled strong echoes.

In 2023, 8 years after the surgery, the suspected malignant lymph nodes of the patient continued to grow, indicating that the iodine 131 treatment was not effective. Her thyroid ultrasound indicated the presence of suspicious lymph nodes in areas II, IV, and VI of the left neck, the largest of which was located in area III, with a size of approximately 3.1 × 2.2 × 0.9 cm. Suspicious lymph nodes were found in areas III, IV, and VI of the right neck, with larger ones exceeding 1 cm in diameter (Fig. 5).

### 2.3. Diagnostic assessment

**Figure 6.**
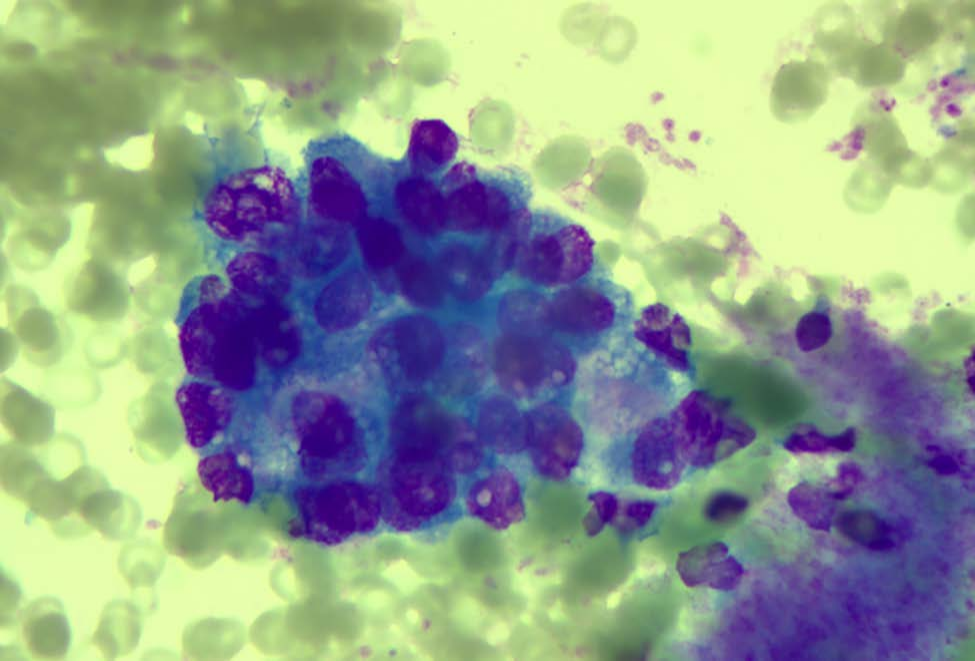
Fine-needle aspiration of the right neck lymph node, revealing cancer cells.

**Figure 7.**
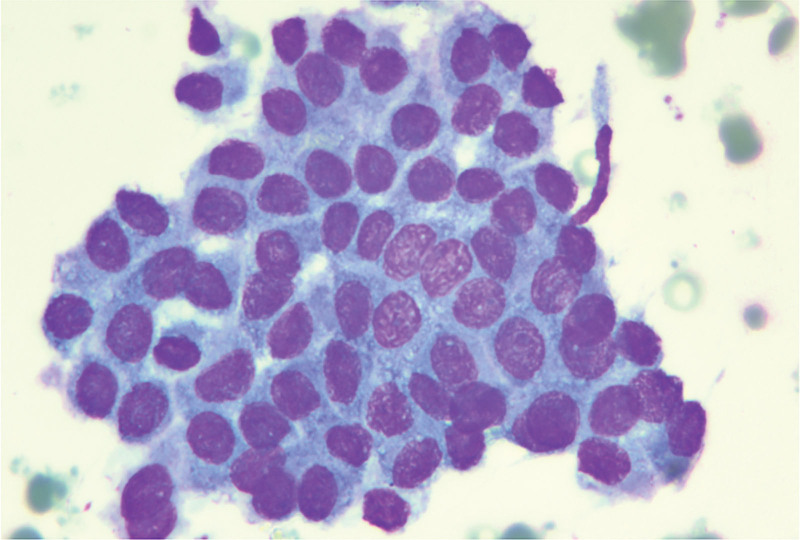
Fine-needle aspiration of lymph nodes in region III of the left neck, revealing cancer cells.

**Figure 8.**
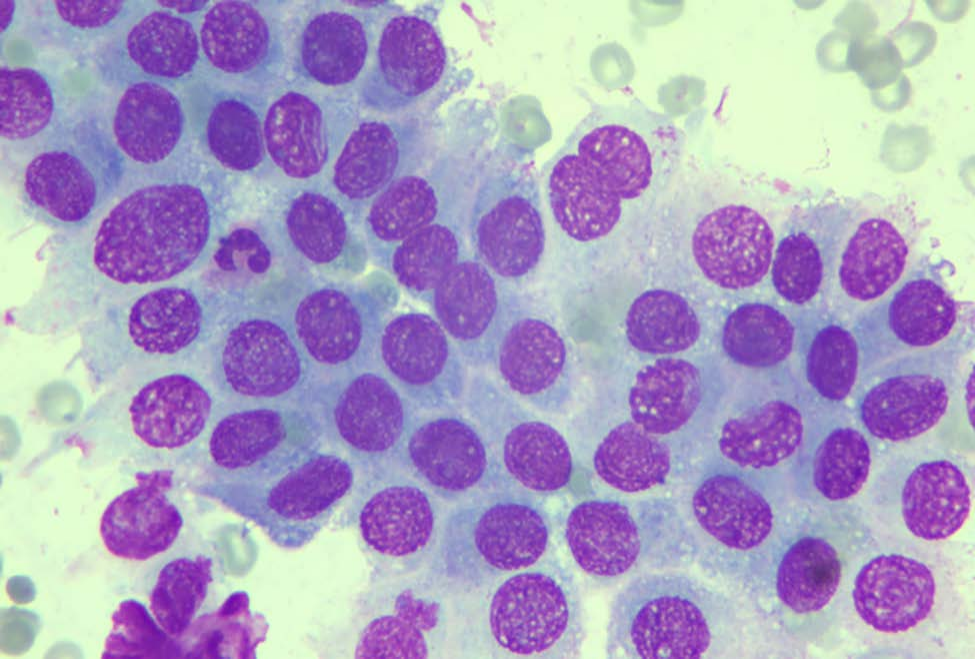
Fine-needle aspiration of lymph nodes in region IV of the left neck revealing cancer cells.

We suggested that the patient undergo fine-needle lymph node puncture, and the puncture results showed the presence of cancer cells in the right cervical lymph nodes, the left neck region III lymph node, and in the IV lymph node of the left neck region, all of which were considered to have a high possibility of thyroid origin (Figs. 6–8).

**Figure 9.**
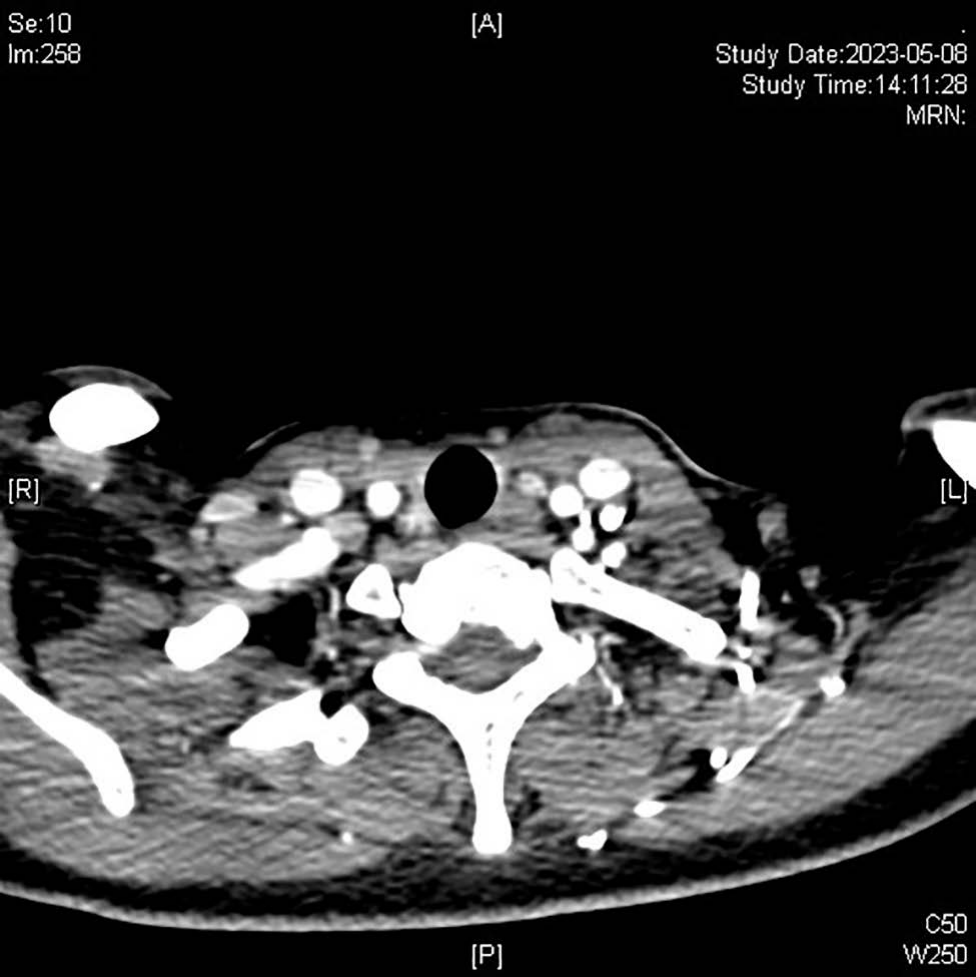
Multiple lymph nodes of varying sizes can be seen on both sides of the neck, with some showing calcification and local fusion. An enhanced scan revealed uneven enhancement and metastasis was suspected.

**Figure 10.**
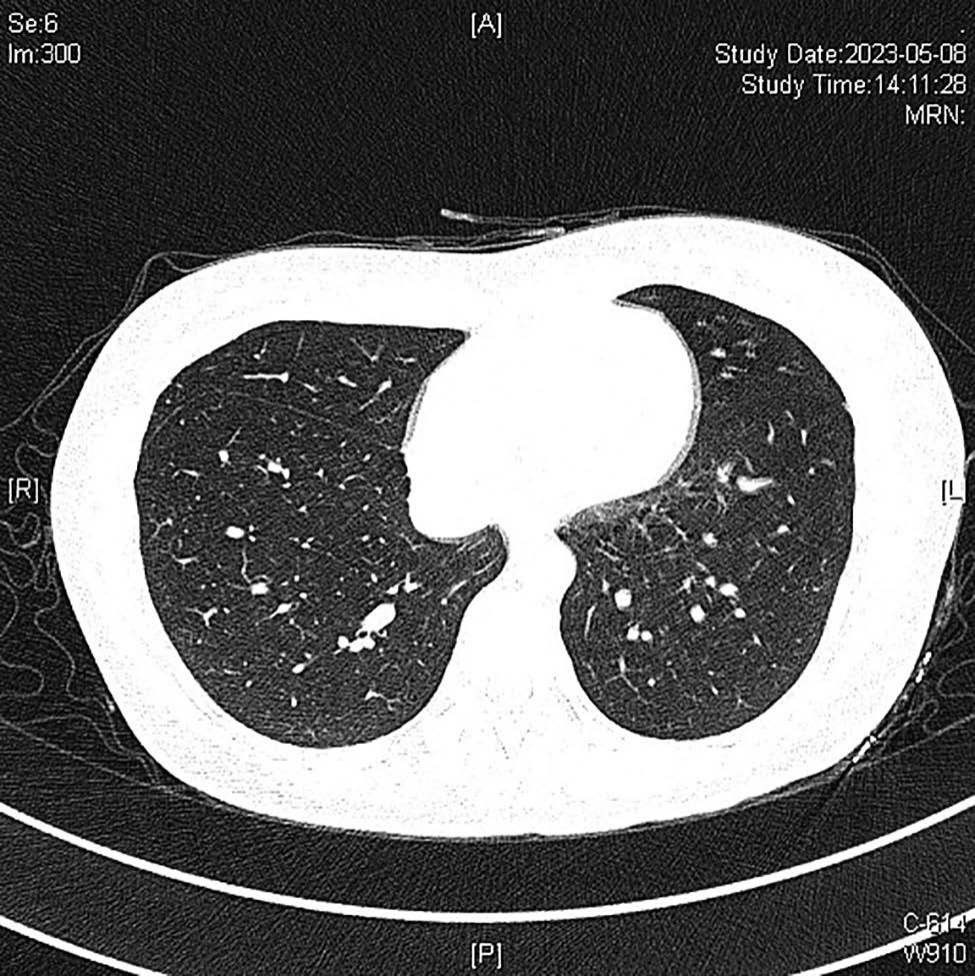
Multiple small nodular lesions in both lungs inclined toward benign nodules.

We then recommended that the patient undergo another surgical treatment, and confirmed that that the patient understood and agreed to the treatment. On physical examination, the neck had palpably enlarged hard lymph nodes with unclear boundaries. The patient free triiodothyronine, free thyroid hormone, anti-thyroid peroxidase antibody, and thyroid-stimulating hormone receptor antibody levels were normal. In contrast, the thyroid-stimulating hormone levels was < 0.017 μIU/mL, and the anti-thyroglobulin antibody level was > 4000 IU/mL, which was significantly higher than normal. A preoperative enhanced CT scan of the patient neck revealed multiple lymph nodes of varying sizes on both sides of the neck, with some showing calcifications (Fig. 9). The larger lesions were located in the deep part of the left sternocleidomastoid muscle (area IIb), with visible local fusion. The cross-section was approximately 1.9 × 1.3 cm, and the enhanced scan showed uneven enhancement, considering transfer. The chest CT results showed multiple small nodular lesions in both lungs inclined toward benign nodules (Fig. 10). Preoperative laryngoscopy revealed no obvious abnormalities, indicating that the tumor had not invaded the trachea.

### 2.4. Therapeutic intervention

The patient underwent bilateral neck lymph node dissection again, and bilateral lymph nodes in areas II, III, and IV were removed. Postoperative paraffin pathology suggested that cancer cells were present in 18 of 35 bilateral cervical lymph nodes. The patient was discharged on the 3rd day after surgery; upon discharge, the patient was generally in good condition. She was prescribed 100 μg oral levothyroxine sodium tablets (to be taken on an empty stomach) every day.

### 2.5. Follow-up and outcomes

**Figure 11.**
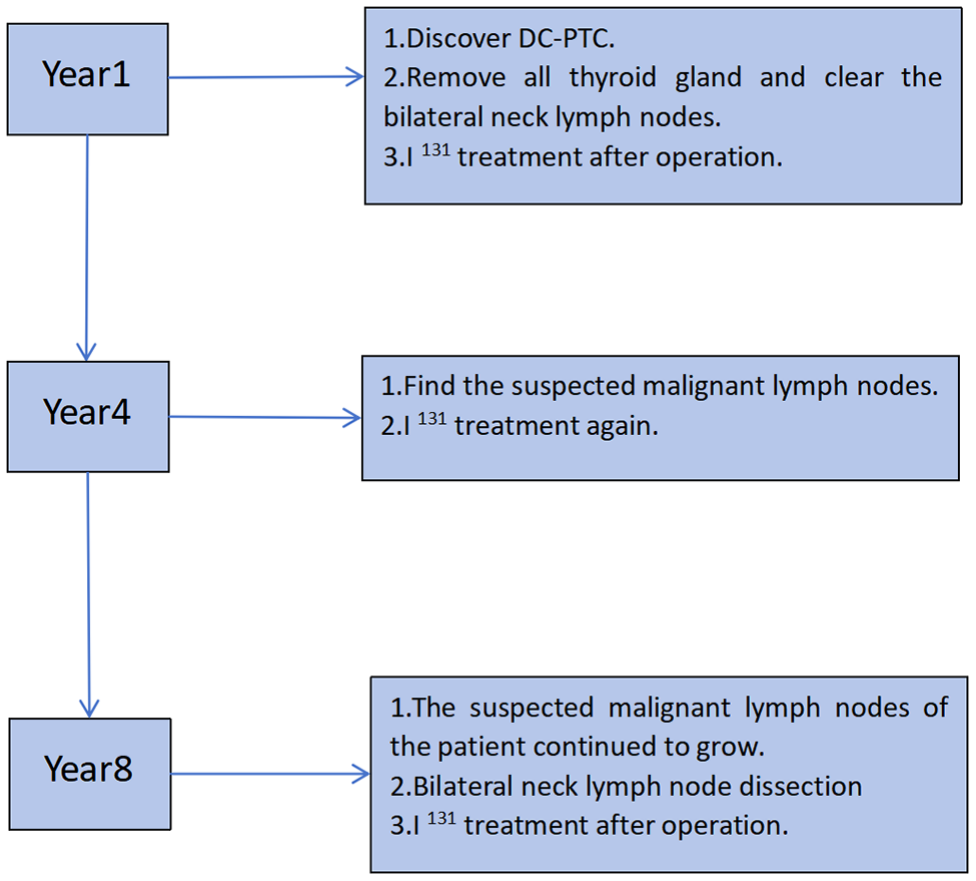
Diagnosis and treatment process.

A follow-up of thyroid function was performed 1 month after surgery and medication dosage was adjusted based on thyroid function results. Two months after surgery, the patient received I131 treatment again at a dose of 110 mCi. Subsequent enhanced CT revealed no residual or focal iodine uptake in the tissue after I131 treatment. Thereafter, the patient underwent regular thyroid ultrasound examinations and no obvious abnormalities were found. In order to better understand the patient diagnosis and treatment process, we have provided a flowchart (Fig. 11).

## 3. Discussion

DSV-PTC is an invasive type of PTC that is relatively rare, accounting for approximately 6% of all PTC cases.^[2]^ It usually occurs in young people, with a male-to-female ratio of approximately 1:4.3. The average age of onset is 28 years, which is significantly earlier than the average age of onset of PTC at 45 years.^[3]^ DSV-PTC is characterized by thyroid enlargement, sclerosis, and diffuse infiltration. It does not usually have obvious nodules but rather exhibits diffuse enlargement of the entire thyroid gland.^[4]^ This type of thyroid cancer is highly invasive and metastatic. It may infiltrate the surrounding tissues and lymph nodes, and metastasize to other parts of the neck or distant organs. Research has shown that the lymph node metastasis rate of DSV-PTC can exceed 80%, which is significantly higher than that of PTC.^[5]^

The symptoms of DSV-PTC include thyroid lumps, neck lumps, hoarseness, difficulty swallowing, neck pain, and enlarged lymph nodes in the neck. Its main histological features are diffuse involvement of the unilateral or bilateral thyroid gland lobes accompanied by dense sclerosis,^[4]^ patchy to dense lymphocyte infiltration, a large number of Sham tumor bodies, extensive squamous metaplasia, and extensive lymphatic infiltration.^[4]^ DSV-PTC is usually diagnosed using thyroid ultrasound, thyroid function examination, fine-needle aspiration biopsy of the thyroid, and pathological examination after surgical resection. The ultrasound image of DSV-PTC shows diffuse distribution of punctate strong echoes, often involving the entire lobe of the gland, which some scholars refer to as a “blizzard” sign. Studies have shown that 17 of 20 patients with DSV-PTC develop HT.^[3]^ Due to the fact that patients with HT also exhibit diffuse lesions and uneven echoes on ultrasound, having HT increases the diagnostic difficulty of DSV-PTC, and DSV-PTC is often misdiagnosed as HT because patients with HT also exhibit diffuse lesions and uneven echoes on ultrasound.

The treatment plan for DSV-PTC remains controversial. Treatment typically involves surgical removal of the thyroid and lymph nodes, as well as iodine 131 radiation therapy and thyroid hormone replacement therapy. American Telemedicine Association^[6]^ suggests that depending on the patient condition, thyroid lobectomy or total thyroidectomy should be performed depending on the patient condition. Radical neck lymph node dissection is required in patients with lymph node metastasis. Based on the postoperative paraffin pathology results, we could further decide whether to undergo iodine 131 treatment. Due to the invasiveness and metastasis of DSV-PTC, its prognosis is usually poor. However, early diagnosis and treatment can improve patient survival. Therefore, regular thyroid examinations and the early detection of lesions are important.

In this case, when the patient first discovered abnormalities, we immediately performed a radical surgery on the patient, removing all the thyroid gland and performing a thorough neck lymph nodes dissection. Facts have proven that this is timely and effective. Four years after the surgery, the patient discovered abnormal enlargement of the neck lymph nodes again. Due to the patient unwillingness to undergo surgical treatment, we performed I 131 treatment on her, which proved to be ineffective. It was not until 8 years after the surgery that the patient underwent the second radical surgery. If the patient can undergo the second surgery in a timely manner, her cervical lymph nodes metastasis may be less.

## 4. Conclusions

DSV-PTC is a rare, invasive subtype of PTC that is more common among young people. It has a high lymph node metastasis rate, high recurrence metastasis rate, and relatively poor prognosis. If DSV-PTC is suspected, imaging examinations such as ultrasound and CT or cytological examinations such as fine-needle aspiration should be performed in a timely manner. Surgical treatment should be performed after the diagnosis. Postoperatively, patients should undergo regular follow-up and lymph node status monitoring. In this article, the patient and her family members are satisfied with our treatment, and she has been persisting in follow-up examinations after surgery. Wishing her good health.

## Author contributions

**Formal analysis:** Jintao Ren, Xuan Zhang.

**Resources:** Chong Geng, Kailin Liu.

**Supervision:** Xingsong Tian.

**Writing – original draft:** Ningning Ren.

**Writing – review & editing:** Ningning Ren.
